# The Casein kinase 1α agonist pyrvinium attenuates Wnt-mediated CK1α degradation *via* interaction with the E3 ubiquitin ligase component Cereblon

**DOI:** 10.1016/j.jbc.2022.102227

**Published:** 2022-07-01

**Authors:** Chen Shen, Anmada Nayak, Leif R. Neitzel, Fan Yang, Bin Li, Charles H. Williams, Charles C. Hong, Yashi Ahmed, Ethan Lee, David J. Robbins

**Affiliations:** 1 Department of Oncology, Lombardi Comprehensive Cancer Center, Georgetown University, Washington, District of Columbia, USA; 2 Molecular Oncology Program, The DeWitt Daughtry Family Department of Surgery, Miller School of Medicine, University of Miami, Miami, Florida, USA; 3 Department of Medicine, University of Maryland, Baltimore, Maryland, USA; 4 Department of Molecular and Systems Biology and the Norris Cotton Cancer Center, Geisel School of Medicine, Dartmouth College, Hanover, New Hampshire, USA; 5 Department of Cell and Developmental Biology, Vanderbilt University, Nashville, Tennessee, USA

**Keywords:** CK1α agonist, CRBN, CK1α, Wnt signaling, immunomodulatory drug, β-Cat S45, β-Catenin S45, CK1α, Casein kinase 1α, CRBN, Cereblon, CRC, colorectal cancer, CRL4^CRBN^, CUL4-RBX-DDB1-CRBN, DMSO, dimethyl sulfoxide, GST, glutathione-*S*-transferase, HEK293T, human embryonic kidney 293T cell line, IMiD, immunomodulatory drug

## Abstract

The Cullin-RING ligase 4 E3 ubiquitin ligase component Cereblon (CRBN) is a well-established target for a class of small molecules termed immunomodulatory drugs (IMiDs). These drugs drive CRBN to modulate the degradation of a number of neosubstrates required for the growth of multiple cancers. Whereas the mechanism underlying the activation of CRBN by IMiDs is well described, the normal physiological regulation of CRBN is poorly understood. We recently showed that CRBN is activated following exposure to Wnt ligands and subsequently mediates the degradation of a subset of physiological substrates. Among the Wnt-dependent substrates of CRBN is Casein kinase 1α (CK1α), a known negative regulator of Wnt signaling. Wnt-mediated degradation of CK1α occurs *via* its association with CRBN at a known IMiD binding pocket. Herein, we demonstrate that a small-molecule CK1α agonist, pyrvinium, directly prevents the Wnt-dependent interaction of CRBN with CK1α, attenuating the consequent CK1α degradation. We further show that pyrvinium disrupts the ability of CRBN to interact with CK1α at the IMiD binding pocket within the CRBN–CK1α complex. Of note, this function of pyrvinium is independent of its previously reported ability to enhance CK1α kinase activity. Furthermore, we also demonstrate that pyrvinium attenuates CRBN-induced Wnt pathway activation *in vivo*. Collectively, these results reveal a novel dual mechanism through which pyrvinium inhibits Wnt signaling by both attenuating the CRBN-mediated destabilization of CK1α and activating CK1α kinase activity.

Casein kinase 1α (CK1α) is a negative regulator of Wnt signaling, participating in the formation of a macromolecular protein complex that mediates the degradation of the Wnt transcription activator β-Catenin (1, 2, 3, 4). Because of this function, CK1α has been shown to be a promising therapeutic target in Wnt-driven cancers such as colorectal cancer (CRC) (5, 6). We previously showed that a class of small molecules potently stimulate the kinase activity of CK1α, resulting in attenuated Wnt activity (7, 8, 9, 10). Importantly, even though these CK1α agonists efficiently inhibit the growth of Wnt-driven CRCs, the Wnt-dependent homeostasis of normal intestinal epithelium remained unperturbed (7, 8, 9). We noted that CK1α levels in tumor tissue are significantly lower than that in normal intestinal tissue (7). Thus, we postulated that the differential abundance of CK1α in distinct tissues is the consequence of its Wnt-dependent regulation, and that the resultant lower levels of CK1α in CRC sensitize tumor tissue to CK1α agonists (7).

Our recent work demonstrated that Wnt signaling induces CK1α degradation *via* a proteosome-dependent mechanism. Furthermore, we showed that this degradation requires the substrate receptor of the CUL4-RBX-DDB1-CRBN (CRL4^CRBN^) E3 ubiquitin ligase complex, Cereblon (CRBN) (11, 12). CRBN has been previously shown to target various proteins for ubiquitination and subsequent degradation (11, 13, 14, 15, 16, 17, 18, 19, 20, 21, 22, 23, 24, 25). The function of the CRL4^CRBN^ complex is modulated by a class of immunomodulatory drugs (IMiDs) that includes thalidomide and lenalidomide (26, 27). This class of small molecules acts as a bridge between CRBN and a set of neosubstrates required for the growth of hematologic tumors, such as multiple myeloma and myelodysplastic syndrome (14, 15, 16, 18, 23, 24, 25, 26, 27, 28, 29). Amongst these CRBN neosubstrates, CK1α is ubiquitinated and degraded in the presence of lenalidomide, which leads to attenuation of the growth of myelodysplastic syndrome with deletion of chromosome 5q (16). However, we showed that, in addition to its role as a lenalidomide-dependent neosubstrate, CK1α is also a physiological substrate of CRBN in response to Wnt signaling (11). Although the Wnt-induced and CRBN-mediated, proteasomal degradation of CK1α occurs in an IMiD-independent fashion (11), we found that the IMiD binding pocket on CRBN–CK1α is structurally important for this regulation (11, 30). Because of its role in degrading CK1α, CRBN promotes Wnt signal transduction in an evolutionarily conserved manner, highlighting CRBN as a novel component of the Wnt signaling pathway (11).

We previously demonstrated that the small-molecule CK1α agonist, pyrvinium (Sigma), inhibits Wnt signaling by enhancing the kinase activity of CK1α (8, 9, 10). Given the newly identified physiological role for CRBN in regulating CK1α stability, we further investigated the effect of this class of small molecules on CRBN-controlled CK1α abundance in the context of Wnt signaling. We show that pyrvinium blocks Wnt-dependent and CRBN-mediated CK1α proteolysis and thus inhibits Wnt pathway activity, revealing a new mechanism of action by which pyrvinium attenuates Wnt signaling through CRBN–CK1α regulation.

## Results

**Figure 1:**
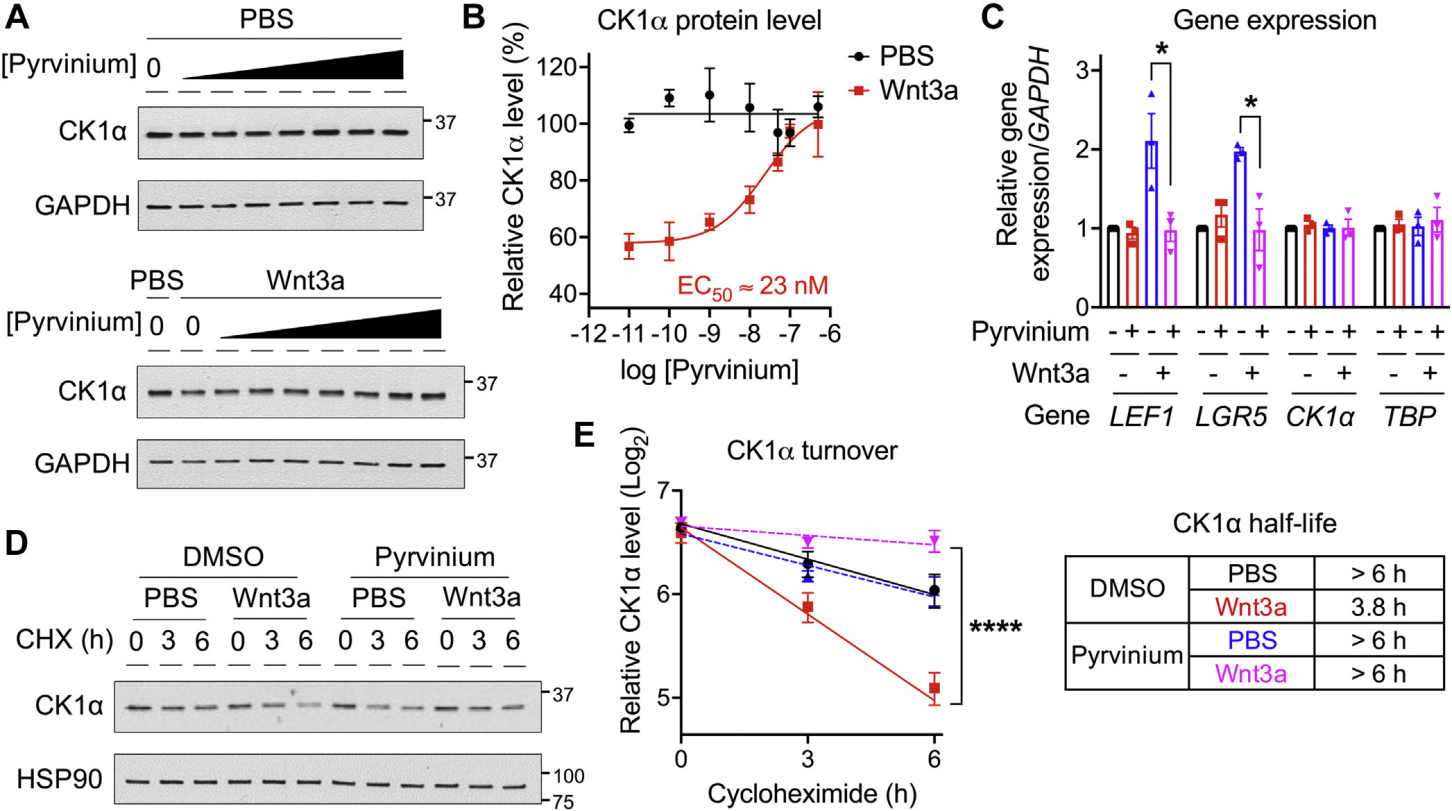
**Wnt-dependent CK1α degradation is inhibited by the CK1α agonist pyrvinium.***A* and *B*, HEK293T cells were treated with recombinant Wnt3a for 24 h, followed by treatment with pyrvinium at various doses for 4 h. Cell lysates were used for immunoblotting, and CK1α protein levels were quantitated and normalized to that of GAPDH. A representative immunoblot (*A*) and the quantification of immunoblots from three independent experiments (*B*) are shown. *C*, HEK293T cells were treated with recombinant Wnt3a for 24 h, followed by treatment with pyrvinium (200 nM) for 4 h. Total RNA was extracted, reverse transcribed to complementary DNA, and used for quantitative RT–PCR analysis. Expression of two Wnt target genes (*LEF1* and *LGR5*), *CK1α*, and *TBP* was quantitated and normalized to that of *GAPDH* (n = 3). Statistical significance is indicated by *asterisks*. *D* and *E*, HEK293T cells were treated with PBS or Wnt3a, along with DMSO or pyrvinium (200 nM), in the presence of cycloheximide for the indicated periods. Cell lysates were used for immunoblotting, and CK1α protein levels were subsequently quantitated and normalized to that of heat shock protein 90 (HSP90). A representative immunoblot (*D*), the quantification of immunoblots from three independent experiments (*E*; *left panel*), and the calculated CK1α half-life under the various conditions (*E*; *right panel*) are shown. Statistical significance is indicated by *asterisks*. HEK293T, human embryonic kidney 293T cell line.

Our previous work showed that during Wnt signaling, CK1α is destabilized by the CRL4^CRBN^ E3 ubiquitin ligase *via* a mechanism analogous to how IMiDs downregulate CK1α (11). As CK1α is reactivated by various small-molecule agonists (7, 8, 9, 10), we speculated that CK1α agonists modulate Wnt- or IMiD-dependent and CRBN-mediated CK1α degradation. To test this hypothesis, we used the Food and Drug Administration–approved anthelmintic drug pyrvinium, which represents the first-in-class CK1α agonist (8), as a tool compound. We first treated human embryonic kidney 293T (HEK293T) cells with recombinant Wnt3a, or PBS, followed by cotreatment with increasing doses of pyrvinium. Consistent with our previous work (11), Wnt3a treatment significantly decreased CK1α levels (Fig. 1, *A* and *B*). Pyrvinium prevented this Wnt-induced CK1α decrease and did so in a dose-dependent manner (Fig. 1, *A* and *B*), with an EC_50_ of approximately 20 nM (Fig. 1*B*). Importantly, while pyrvinium (200 nM) was able to attenuate this Wnt-dependent decrease in CK1α levels (Fig. 1, *A* and *B*), it did not alter the gene expression level of *CK1α* (Fig. 1*C*). We further investigated the turnover of CK1α in the presence of pyrvinium using a cycloheximide chase assay (11) and noted that pyrvinium significantly prolonged the half-life of CK1α in the presence of Wnt3a (Fig. 1, *D* and *E*). These results indicate that pyrvinium inhibits the Wnt-dependent degradation of CK1α (11).

**Figure 2:**
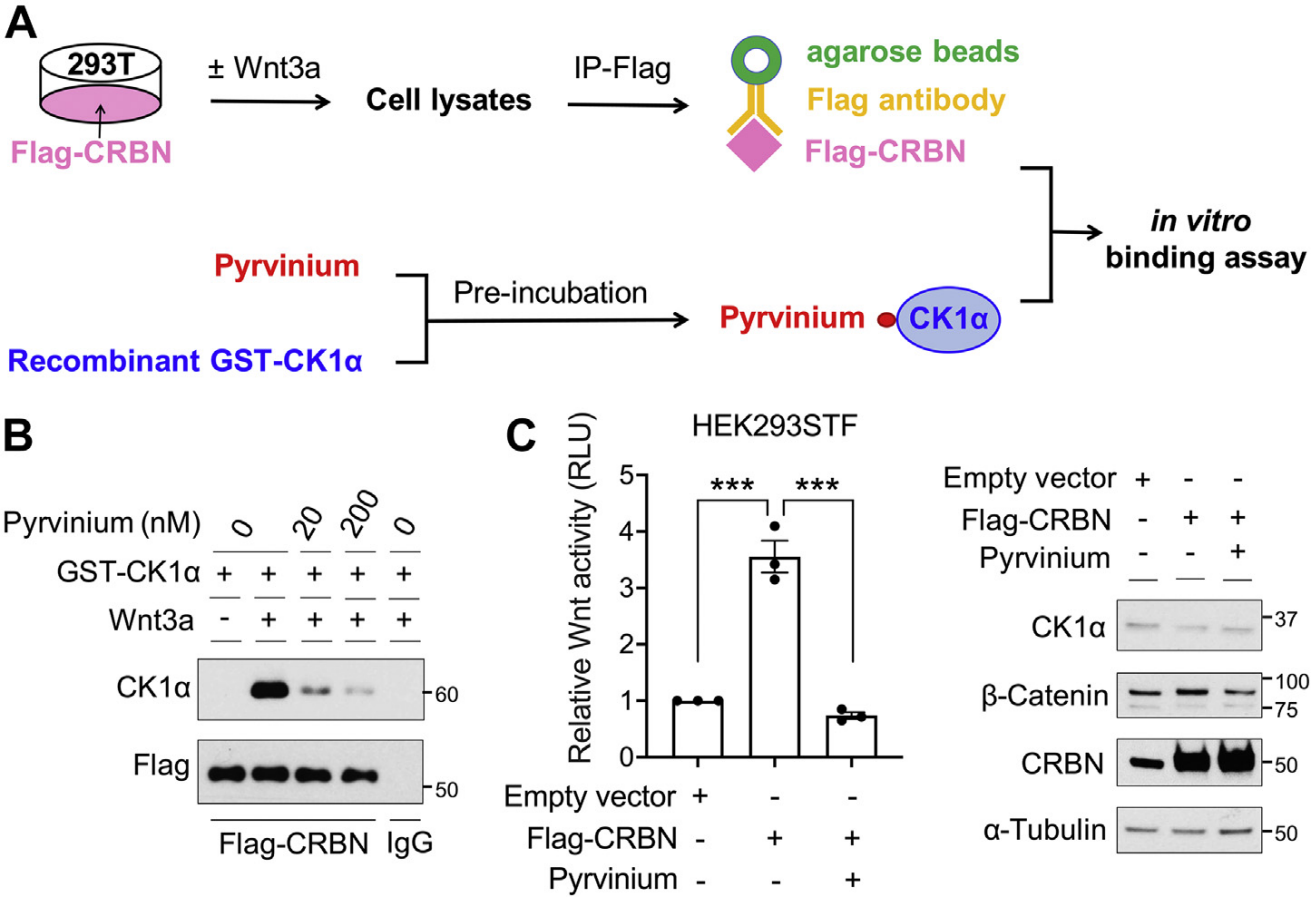
**Pyrvinium disrupts Wnt-driven CRBN–CK1α association and activity.***A*, a schematic showing the workflow of the *in vitro* CRBN–CK1α binding assay used in (*B*). *B*, HEK293T cells were transfected with a plasmid encoding FLAG-tagged CRBN, followed by PBS or Wnt3a treatment. FLAG-CRBN was immunopurified from these cell lysates, and a mixture of recombinant GST-tagged CK1α and DMSO or pyrvinium incubated together. CK1α bound to FLAG-CRBN was eluted by Laemmli sample buffer and analyzed by immunoblotting. A representative immunoblot is shown (n = 3). *C*, HEK293STF Wnt reporter cells were transfected with a plasmid encoding FLAG-tagged CRBN or a control vector, followed by treatment with pyrvinium (200 nM) for 4 h. Cell lysates were used for luciferase assay or immunoblotting. Luminescence signals were normalized to total protein concentration and indicated as Wnt reporter activity. The quantification of Wnt reporter activity from three independent experiments (*left panel*) and a representative immunoblot (*right panel*) are shown. Statistical significance is indicated by *asterisks*. CRBN, Cereblon; DMSO, dimethyl sulfoxide; GST, glutathione-*S*-transferase; HEK293T, human embryonic kidney 293T cell line.

Wnt pathway activation induces CRBN to bind and ubiquitinate CK1α, leading to CK1α proteolysis (11). Thus, we next studied the effect of pyrvinium on the Wnt-driven association of CRBN and CK1α using an *in vitro*–binding assay. We incubated CRBN isolated from Wnt3a- or PBS-treated cells and recombinant CK1α protein in the presence or the absence of pyrvinium, followed by immunoblotting to detect CRBN-bound CK1α (Fig. 2*A*). We found that nanomolar amounts of pyrvinium significantly decreased the binding of recombinant CK1α to Wnt-modulated CRBN (Fig. 2*B*), suggesting that pyrvinium is capable of directly preventing the recruitment of CRBN to CK1α.

We previously showed that pyrvinium acts as a Wnt inhibitor by stimulating CK1α kinase activity (7, 8, 9, 10). Nevertheless, our current results suggest that pyrvinium may also attenuate Wnt signal transduction by increasing CK1α stability. We therefore determined the capacity of pyrvinium to inhibit Wnt activity driven by CRBN-dependent CK1α degradation. Overexpression of CRBN in HEK293STF (SuperTopFlash) cells, which express a Wnt-driven TOPFlash transcriptional reporter, significantly reduced CK1α levels (Fig. 2*C*, *right* *panel*) and increased the activity of this Wnt reporter gene (Fig. 2*C*, *left* *panel*). Notably, the addition of pyrvinium attenuated the CRBN-dependent reduction in CK1α levels (Fig. 2*C*, *right* *panel*) and subsequently blocked CRBN-driven Wnt reporter activation (Fig. 2*C*, *left* *panel*). Collectively, these results support the conclusion that pyrvinium can also attenuate Wnt pathway activity by reducing the ability of CRBN to associate with CK1α and consequently preventing CRBN-mediated degradation of CK1α.

**Figure 3:**
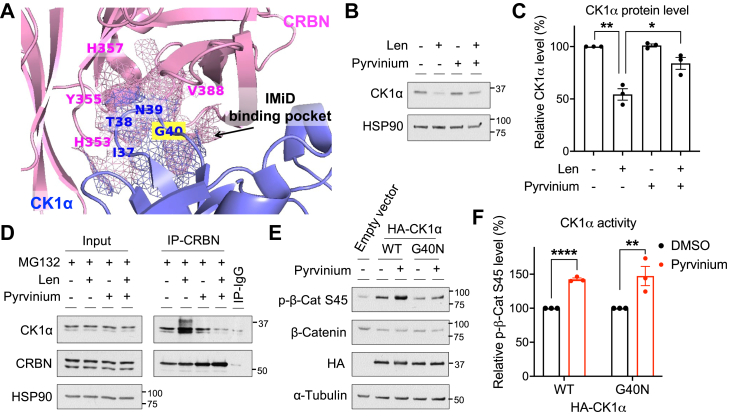
**Pyrvinium inhibits CK1α degradation *via* the CRBN–CK1α IMiD binding pocket.***A*, a structural model of CK1α–CRBN complex upon Wnt activation is shown (Protein Data Bank ID: 5FQD; model generated using PyMOL 2.5 software). Some crucial residues in the IMiD binding pocket (shown in *mesh*) are as labeled. *B* and *C*, HEK293T cells were treated with DMSO or lenalidomide (10 μM) for 4 h and subsequently with DMSO or pyrvinium (200 nM) for another 4 h. Cell lysates were used for immunoblotting analysis. A representative immunoblot (*B*) and the quantification of immunoblots from three independent experiments (*C*) are shown. Statistical significance is indicated by *asterisks*. *D*, HEK293T cells were treated with DMSO or lenalidomide (10 μM) for 4 h and subsequently with DMSO or pyrvinium (200 nM) for another 4 h in the presence of MG132 (10 μM). Cell lysates were used for CRBN immunoprecipitation. Proteins bound to CRBN were eluted by Laemmli sample buffer and analyzed by immunoblotting. A representative immunoblot is shown (n = 3). *E* and *F*, HEK293T cells were transfected with a plasmid encoding hemagglutinin (HA)-tagged WT CK1α or CK1α G40N mutant or a control vector, followed by treatment of pyrvinium (200 nM) for 30 min. Cell lysates were used for immunoblotting analysis. A representative immunoblot (*E*) and the quantification of immunoblots from three independent experiments (*F*) are shown. Statistical significance is indicated by *asterisks*. CRBN, Cereblon; DMSO, dimethyl sulfoxide; IMiD, immunomodulatory drug.

The ability of CRBN to destabilize CK1α requires a pocket within the CRBN–CK1α complex to which IMiDs bind (Fig. 3*A*), even though this regulation does not require binding of an IMiD (11, 30). Hence, we hypothesized that this IMiD binding pocket might also be required for pyrvinium’s ability to inhibit CRBN-modulated CK1α degradation. To test this hypothesis, we treated HEK293T cells with the IMiD, lenalidomide (Sigma), which regulates CK1α levels *via* this pocket (11), followed by incubation with or without pyrvinium. Lenalidomide induced a significant decrease in CK1α levels (Fig. 3, *B* and *C*) as previously reported (11, 16), and this activity was attenuated in the presence of pyrvinium (Fig. 3, *B* and *C*). We further showed that pyrvinium disrupts the previously described neomorphic binding of CRBN to CK1α that is bridged by lenalidomide (Fig. 3*D*). Together, these results suggest that pyrvinium inhibits CRBN-modulated CK1α proteolysis *via* the IMiD binding pocket.

We next compared the kinase activity of WT CK1α and a CK1α mutant (G40N) in which the IMiD binding pocket of CRBN–CK1α is disrupted (11, 30) (Fig. 3*A*, residue highlighted in *yellow*), to determine if pyrvinium requires this IMiD binding pocket to activate CK1α kinase activity. HEK293T cells expressing WT or G40N CK1α were treated with pyrvinium or dimethyl sulfoxide (DMSO), and the levels of phosphorylated β-Catenin S45 (β-Cat S45), an endogenous CK1α-specific substrate (2, 3, 10), was monitored by immunoblotting. We found that the kinase activity of both CK1α WT and G40N toward β-Cat S45 was enhanced by pyrvinium to a similar extent (Fig. 3, *E* and *F*). This result suggests that the IMiD binding pocket of CRBN–CK1α is not required for pyrvinium to stimulate CK1α kinase activity, despite its importance for blocking CRBN-mediated CK1α binding and degradation by pyrvinium (Fig. 3, *B*–*D*).

**Figure 4:**
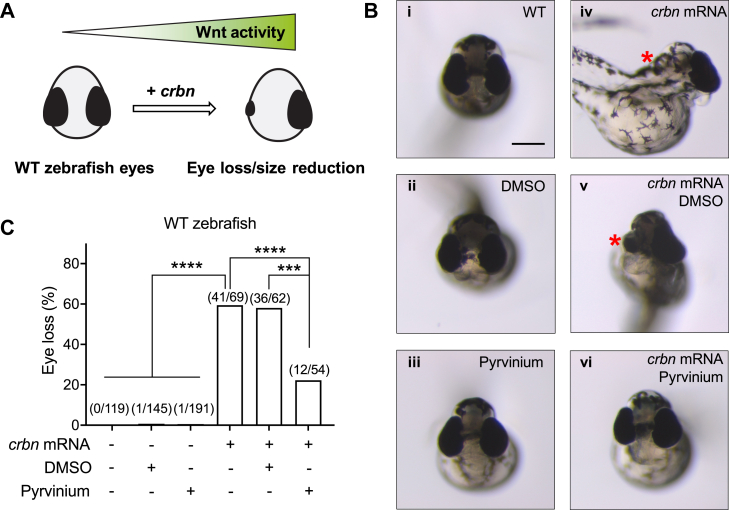
**Pyrvinium attenuates CRBN-driven Wnt activity *in vivo*.***A*, a schematic of zebrafish eye phenotype in response to CRBN-driven Wnt activation. *B*, WT zebrafish (i) or zebrafish injected with *crbn* mRNA (iv) were treated with DMSO (ii and v) or pyrvinium (iii and vi). Transverse views are shown (the scale bar represents 200 μm). Lost or reduced eye is indicated by a *red asterisk*. *C*, quantification of zebrafish with eye loss/reduction from (*B*). Statistical significance is indicated by *asterisks*. CRBN, Cereblon; DMSO, dimethyl sulfoxide.

Wnt signaling plays an essential role in the development of numerous model organisms including the zebrafish *Danio rerio*, in which aberrant activation of Wnt signaling leads to the loss of the eyes (31) (Fig. 4*A*). To investigate the effect of pyrvinium on CRBN-driven Wnt activity *in vivo*, we injected *crbn* mRNA into zebrafish embryos, followed by treatment with pyrvinium or DMSO, and evaluated the eye phenotype. We found that overexpression of *crbn* in zebrafish induced significant eye loss (59.4%) (Fig. 4, *B*iv and *C*) relative to controls (Fig. 4, *B*i and *C*), consistent with our previous findings that Crbn positively modulates Wnt signaling *in vivo* (11) (Fig. 4*A*). Importantly, this Crbn-driven Wnt-dependent eye loss was significantly reduced by pyrvinium treatment (22.2%) (Fig. 4, *B*vi and *C*) relative to the vehicle control (58.1%) (Fig. 4, *B*v and *C*). Of note, neither pyrvinium treatment nor DMSO treatment led to eye loss in WT embryos (Fig. 4, *B*ii and iii and *C*), supporting the specific effect of pyrvinium on *crbn* mRNA–induced eye phenotype. These results suggest that pyrvinium is capable of inhibiting CRBN-driven Wnt pathway activation *in vivo*. Taken together, these findings show that pyrvinium is capable of attenuating the evolutionarily conserved function of CRBN in the activation of Wnt signaling.

## Discussion

**Figure 5:**
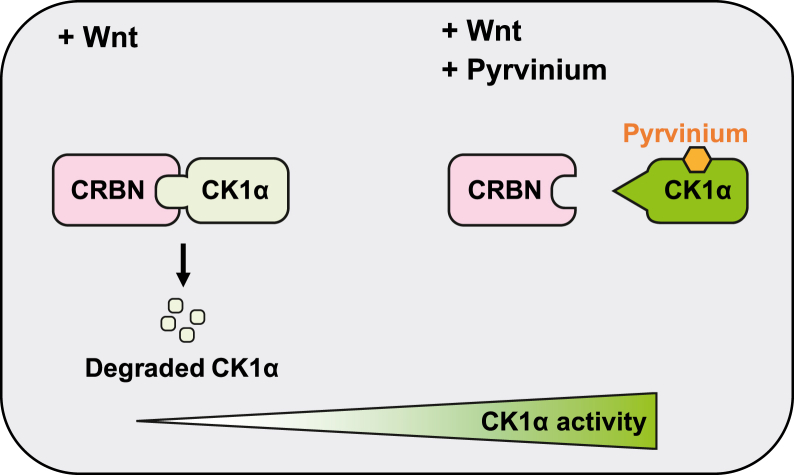
**A model depicting pyrvinium’s dual mechanism of CK1α activation.** In response to Wnt pathway activation, CRBN binds to CK1α in the IMiD binding pocket and induces CK1α degradation. However, upon binding by pyrvinium, CK1α undergoes allosteric regulation, which inhibits its Wnt-dependent association with CRBN and consequently blocks its degradation. This modulation also leads to the stimulation of CK1α kinase activity, albeit independently of CK1α stabilization. CRBN, Cereblon; IMiD, immunomodulatory drug.

Herein, we show that in addition to its known role in activating CK1α kinase activity (7, 8, 9, 10), pyrvinium stabilizes CK1α by directly preventing its interaction with the E3 ubiquitin ligase component CRBN (Fig. 5). Furthermore, in contrast with its ability to activate the kinase activity of CK1α, pyrvinium’s ability to inhibit CRBN-mediated CK1α degradation requires the IMiD binding pocket within the CRBN–CK1α complex. Importantly, pyrvinium also rescues Wnt-activated phenotypes induced by CRBN overexpression *in vivo*.

While many drugs have been shown to link CRL4^CRBN^ E3 ubiquitin ligase and substrates (26), we describe here for the first time a small molecule that can disrupt the binding of CRL4^CRBN^ to its substrate (CK1α) and thereby inhibiting CRBN-targeted proteolysis. In this process, the IMiD binding pocket of CRBN–CK1α is critical. Disruption of the structure of this pocket by mutating one of the key residues (CK1α G40N) inhibits both the association of CK1α with CRBN and its degradation (11, 30). However, pyrvinium retains the ability to activate CK1α G40N (Fig. 3, *E* and *F*). This observation suggests that the inhibition of CK1α degradation and the stimulation of CK1α kinase activity by pyrvinium are mediated by independent mechanisms. Whereas the exact details remain unknown, we propose that binding of pyrvinium to CK1α allosterically regulates the catalytic capacity of CK1α (Fig. 5), consistent with our previous data showing that pyrvinium increases the *V*_max_ of CK1α but not its *K*_*m*_ (10). This conformational regulation may be related with post-translational modifications of CK1α, such as autophosphorylation (32). In addition, pyrvinium’s binding to CK1α may also lead to an alteration in the structure of its N-terminal β-hairpin loop, reducing its association with CRBN and subsequent degradation (Fig. 5).

CK1α agonists are potent inhibitors of Wnt activity and Wnt-driven CRC growth, with IC_50_s in the low nanomolar range (7, 8, 9, 10). We noted that these small molecules induce an approximate twofold activation of CK1α kinase activity *in vitro* (7, 8, 9, 10), which is a lower level of activation than a number of other types of kinase activators (33). Furthermore, the effects of pyrvinium on CK1α stability and on Wnt target gene expression appear to be Wnt dependent. Thus, we anticipate that the potent Wnt-inhibiting effect of CK1α agonists might result from their dual function in both stabilizing CK1α and then activating this stabilized pool of protein. CK1α agonists have also shown antitumor efficacy *via* a number of other mechanisms besides inhibition of Wnt signaling (34, 35, 36). Thus, this study also sheds light on the potential role of a CRBN–CK1α regulatory axis in controlling other signaling pathways, such as Hedgehog signaling (35) and autophagy (36). Taken together, this work demonstrates that, in addition to its conventional role in enhancing CK1α catalytic efficiency, pyrvinium also attenuates Wnt signaling by regulating the function of CRBN.

## Experimental procedures

### Reagents

Recombinant human Wnt3a (R&D Systems) was reconstituted and stored as per the manufacturer’s instructions. Cycloheximide (EMD Millipore) was dissolved in DMSO to 50 mg/ml. Pyrvinium, lenalidomide, and MG132 (Selleck Chemicals) were dissolved in DMSO to 10 mM. All drug stocks were stored at −20 °C. A plasmid encoding zebrafish Crbn was synthesized in pCS2 vector (Addgene) and that encoding human hemagglutinin-tagged CK1α (NP_001883.4) and FLAG-tagged CRBN (NP_001166953.1) were synthesized in pcDNA3.1(+) vector (Addgene) and site-directed mutated by GenScript. Recombinant human glutathione-*S*-transferase (GST)-CK1α protein was purified from Sf9 insect cells expressing a plasmid encoding GST-CK1α (NP_001883.4) (GenScript).

### Cellular assays

HEK293T and HEK293STF cells were purchased from American Type Culture Collection and cultured as recommended. Recombinant Wnt3a was added to 50 to 70% confluent cells at 250 ng/ml for 24 h if not otherwise specified. Cycloheximide was added to cells at a concentration of 50 μg/ml. Quantitative PCR was performed as previously described (7, 11) using TaqMan probes (Applied Biosystems) targeting indicated genes. Plasmids were transfected using Lipofectamine 2000 (Invitrogen) for 48 h as per the manufacturer’s instructions. Wnt reporter assay was performed as previously described (7, 11).

### Immunoblotting and immunoprecipitation

CRBN immunoprecipitations were performed as previously described (11). Proteins precipitated were eluted in 4× Laemmli buffer with 2-mercaptoethanol and used for immunoblotting. For immunoblotting, cells were lysed in 2× Laemmli buffer with 2-mercaptoethanol, and the lysates were run on an SDS-PAGE electrophoresis, followed by protein transfer. The primary antibodies used for immunoblotting include CK1α (Abcam); GAPDH, β-catenin, phosphorylated β-cat S45, and hemagglutinin (Cell Signaling Technology); heat shock protein 90 (HSP90; Santa Cruz Biotechnology); FLAG (Sigma); CRBN (Novus Biologicals); and α-tubulin (EMD Millipore). The secondary antibodies used were horseradish peroxidase–conjugated donkey antimouse or anti-rabbit (Jackson ImmunoResearch). Immunoblots were developed using X-ray films. Chemiluminescence of immunoblots was analyzed by ImageJ software (National Institutes of Health).

### *In vitro* binding assay

HEK293T cells were transfected with plasmids encoding an empty vector or FLAG-CRBN, followed by treatment with PBS or Wnt3a for 4 h. FLAG-CRBN was isolated from the lysates of these cells by immunoprecipitation (11). Recombinant GST-CK1α protein was preincubated with pyrvinium in binding buffer (11) at 4 °C for 10 min. The mixtures of GST-CK1α and pyrvinium were then added to bovine serum albumin–blocked FLAG-CRBN–bound beads (11) and incubated in binding buffer at 4 °C for 1 h, followed by one 5-min wash (11) and elution of CRBN-bound CK1α using 2× Laemmli buffer with 2-mercaptoethanol.

### Zebrafish studies

NHGRI-1 zebrafish embryos (one cell) were injected with 1 ng *crbn* mRNA (37) in 1 nl in the single cell. For DMSO and pyrvinium treatments, embryos were incubated at 28.5 °C until 50% epiboly (5.25 h postfertilization) and then soaked in E3 medium with DMSO or pyrvinium (100 μM) until 30 h postfertilization. Embryos were raised, fixed at 3 days postfertilization, and phenotyped (38). Bright field images of embryos were acquired using a Zeiss Stemi 2000-CS microscope with an Olympus DP72 camera. All zebrafish studies were approved by the University of Maryland’s Institutional Animal Care and Use Committee.

### Statistics

All cellular experiments were performed independently for at least three times. The error bars in the quantifications indicate mean ± SEM. *p* Values were determined using two-tailed Student’s *t* test, two-way ANOVA (cycloheximide assay), or Fisher’s exact test (zebrafish studies). *Asterisks* indicate statistical significance (∗*p* < 0.05, ∗∗*p* < 0.01, ∗∗∗*p* < 0.001, and ∗∗∗∗*p* < 0.0001).

## Data availability

All data supporting the findings of this study are available in the article. All other relevant data are available from the authors upon reasonable request.

## Conflict of interest

D. J. R. and E. L. are founders of StemSynergy Therapeutics, Inc, a company commercializing small-molecule signaling inhibitors, including Wnt inhibitors. All other authors declare that they have no conflicts of interest with the contents of this article.
